# A Mechanism Study of Redox Reactions of the Ruthenium-oxo-polypyridyl Complex

**DOI:** 10.3390/molecules28114401

**Published:** 2023-05-28

**Authors:** Bao-Long Chen, Sheng-Yi Yan, Xiao-Qing Zhu

**Affiliations:** The State Key Laboratory of Elemento-Organic Chemistry, Collaborative Innovation Center of Chemical Science and Engineering, College of Chemistry, Nankai University, Tianjin 300071, China; supermansheng@163.com

**Keywords:** Ruthenium-oxo-polypyridyl complex, hydride transfer, kinetic, thermodynamic

## Abstract

Over the years, Ru^IV^(bpy)_2_(py)(O)^2+^([Ru^IV^O]^2+^) has garnered considerable interest owing to its extensive use as a polypyridine mono-oxygen complex. However, as the active-site Ru=O bond changes during the oxidation process, [Ru^IV^O]^2+^ can be used to simulate the reactions of various high-priced metallic oxides. In order to elucidate the hydrogen element transfer process between the Ruthenium-oxo-polypyridyl complex and organic hydride donor, the current study reports on the synthesis of [Ru^IV^O]^2+^, a polypyridine mono-oxygen complex, in addition to **1H** and **3H** (organic hydride compounds) and **1H** derivative: **2**. Through ^1^H-NMR analysis and thermodynamics- and kinetics-based assessments, we collected data on [Ru^IV^O]^2+^ and two organic hydride donors and their corresponding intermediates and established a thermodynamic platform. It was confirmed that a one-step hydride transfer reaction between [Ru^IV^O]^2+^ and these organic hydride donors occurs, and here, the advantages and nature of the new mechanism approach are revealed. Accordingly, these findings can considerably contribute to the better application of the compound in theoretical research and organic synthesis.

## 1. Introduction

As a stoichiometric and catalytic oxidizing agent for a range of organic compounds, Ru^IV^(bpy)_2_(py)(O)^2+^ ([Ru^IV^O]^2+^) has garnered considerable research interest [1]. To date, [Ru**^IV^**O]**^2^**^+^ has been used in the epoxidation of olefins [2]; the oxidation of sulfides to sulfoxide [3,4]; the oxidation of alcohols [5,6], benzyl, and allyl C–H bonds; and the novel oxidation [7] of C–H bonds by hydrogen atom abstraction for alcohols, aldehydes, cycloketones, benzyl, or allyl positions [8,9]. A cursory examination of the literature shows that [Ru**^IV^**O]**^2^**^+^ has the following advantages: (1) Because the synthesis methods have been reported many times, ligands bonded with this agent can be altered to preserve the oxidation active center, Ru^IV^=O, so that their reduction potential can be varied accordingly. (2) Due to their stability, the resultant complexes have been used as electrocatalytic oxidants for various compounds in aqueous solutions [10]. (3) Their physical and chemical properties, as well as their spectral properties [11,12], have been comprehensively investigated [13,14,15,16].

In addition, [Ru^IV^O]^2+^ modifies the active Ru=O bond in oxidation reactions; hence, it can be used to simulate various high-valence Ru oxides. The oxidation of the metallic porphyrin oxide carbonylruthenium(II) meso-tetrakis(pentafluorophenyl) porphyrin [Ru(TPFPP)(CO)] is an example [17]. Serving as a benchmark for the oxidation processes of other high-valence metal oxides, the cytochrome P450 oxidase core model has been proven to be highly effective [18]. Accordingly, quantitative studies of [Ru^IV^O]^2+^ employing chemical reaction kinetics and thermodynamics may prove to be feasible and beneficial. However, such reports are greatly limited, warranting further investigations into the above reaction mechanism.

**Scheme 1:**
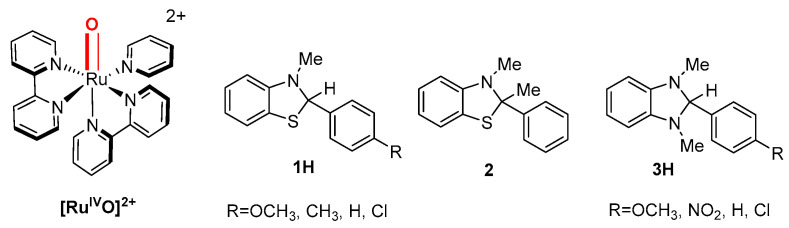
Compounds synthesized herein.

Which compound can reduce high-valence organometallic oxides? We considered organic hydride compounds. The current study reports on the synthesis of two series of hydride compounds, which are shown in Scheme 1. First, proton nuclear magnetic resonance (^1^H-NMR) spectrometry, mass spectrometry, and titration were used to identify the products and stoichiometric ratio of the two types of reactions. Subsequently, the energy change in each elementary reaction was investigated by measuring the corresponding thermodynamic parameters. In addition, the kinetic parameters of the reactions were determined to ascertain the precise path of hydrogen transfer. Thus, these findings serve as a theoretical foundation for the reaction mechanism of the Ruthenium-oxo-polypyridyl complex.

**Scheme 2:**
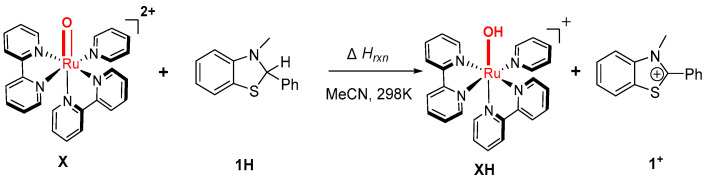
Hydride transfer reactions observed herein.

Free hydride ion is unavailable in acetonitrile. Additionally, the process of [Ru^IV^O]^2+^ acceptance of a hydride anion is contrary to the heterolytic process for producing an anion (Equation (1)). Correspondingly, the thermodynamic driving force of hydride transfer depends on the ability of the hydride donor to provide the hydride, as well as that of the hydride acceptor to capture the hydride (Scheme 2). Nevertheless, the hydride affinity of X in solution, ∆*H*_H−A_(X), can be derived from the reaction enthalpy change of the corresponding carbanions X^+^ with a suitable hydride donor, such as 2,3-dihydrobenzo-[d]thiazoles (1H) (Equation (2)).

∆*H*_H−A_(X) = −∆*H*_H−D_(XH), (1)

∆*H*_r×n_ = ∆*H*_H−A_([Ru^IV^O]^2+^) + ∆*H*_H−D_(XH). (2)

Because the hydride ion includes a proton and two electrons, the hydride transfer process comprises the following paths: a one-step hydride process; a hydrogen–electron transfer process; and an e-H+-e or e-H• multistep transfer mechanism induced by electrons. Subsequently, the process mainly involves the following changes in bond energy: The reaction enthalpy change of [Ru^IV^O]^2+^ to accept hydride ions (hydride affinity), ∆*H*_H−A_([Ru^IV^O]^2+^); the enthalpy change of [Ru^IV^O]^2+^ to accept hydrogen atoms (hydrogen affinity), ∆*H*_HA_([Ru^IV^O]^2+^); the reaction enthalpy change of [Ru^III^O]^+^ to accept hydrogen atoms (hydrogen affinity), ∆*H*_HA_([Ru^III^O]^+^); and the reaction enthalpy change of [Ru^III^O]^+^ to accept protons (proton affinity), ∆*H*_PA_([Ru^III^O]^+^).

∆*H*_HA_([Ru^IV^O]^2+^) = ∆*H*_H−A_ ([Ru^IV^O]^2+^) − F [*E*°(H^0/−^) – *E*°(RuOH^+/2+^)] (3)

∆*H*_PA_([Ru^III^O]^+^) = ∆*H*_HA_ ([Ru^IV^O]^2+^) – F [*E*°(H^+/0^) – *E*°(RuO^+/2+^)] (4)

∆*H*_HA_([Ru^III^O]^+^) = ∆*H*_PA_ ([Ru^III^O]^+^) – F [*E*°(H^+/0^) – *E*°(RuOH^+/2+^)] (5)

**Scheme 3:**
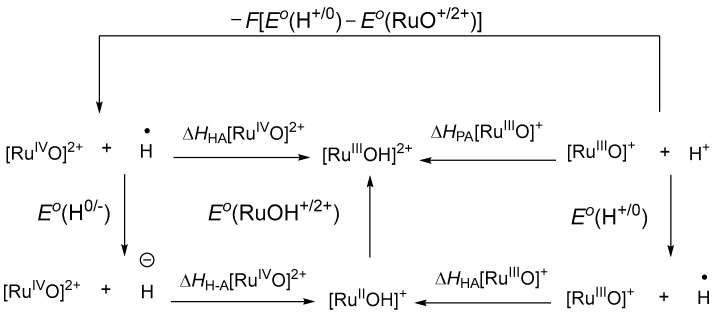
Thermodynamic cycles constructed on the basis of the chemical process of [Ru^IV^O]^2+^ to obtain a hydride anion in acetonitrile at 298 K.

The thermodynamic parameters of the reactants and reaction intermediates (Scheme 3) may be detected on the basis of the electrochemical and calorimetric data; the corresponding derivation equations are shown in Equations (3)–(5). *E*°_red_(X) is the reduction potential of the hydride acceptor compound, whereas *E*°_ox_(XH) is the oxidation potential of the hydride donor compound (Scheme 3). We replaced ∆*H* with ∆*G*_ET_ and referred to the literature value [19] to determine the reversible potentials, taking *E*_1/2_(H^+/0^) = −2.307 (V vs. Fc^+/0^) and *E*_1/2_(H^0/−^) = −1.137 (V vs. Fc^+/0^) (Fc = ferrocene). Correspondingly, the equations shown above can be simplified to Equations (6)–(8). F stands for Faraday’s constant (23.05 kcal mol**^−^**^1^V**^−^**^1^).

∆*H*_HA_ (X) = ∆*H*_H_−_A_ (X) – F [*E*°(H^0/−^) – *E*°_ox_(XH)] (6)

∆*H*_PA_(X^•−^) = ∆*H*_HA_ (X)− F [*E*°(H^+/0^) – *E*°_red_(X)] (7)

∆*H*_HA_(X^•−^) = ∆*H*_PA_ (X^•−^) – F [*E*°(H^+/0^) – *E*°_ox_(XH)] (8)

## 2. Results

### 2.1. Results for the Reaction of Ruthenium-oxo-polypyridyl Compounds and ***1H***

**Figure 1:**
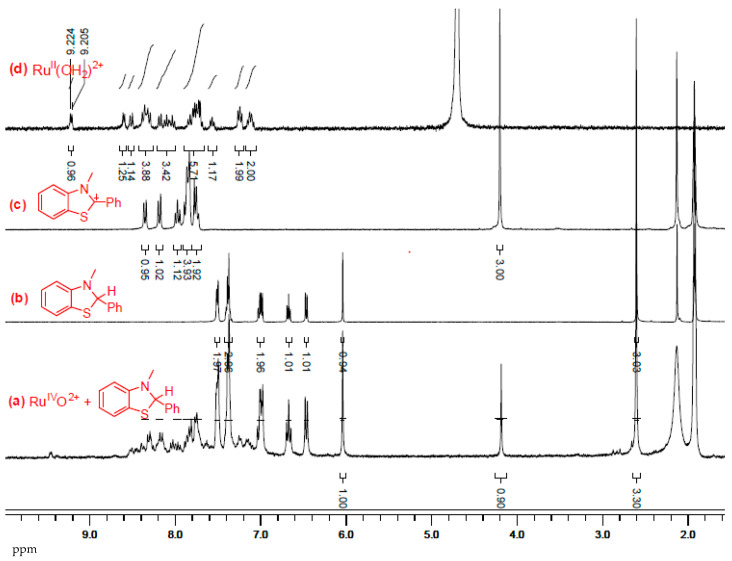
Overlap in the ^1^H-NMR spectra of (**a**) the reaction mixture in CD_3_CN containing [Ru**^IV^**O]**^2^**^+^ (3.01 mM) with **1H**(R=H) (12.04 mM) after 30 min, (**b**) **1H**(R=H) in CD_3_CN, (**c**) 3-methyl-2-phenylbenzo[d]thiazol-3-ium in CD_3_CN, and (**d**) Ru^II^(bpy)_2_(py)(OH_2_)^2+^ in D_2_O.

**Scheme 4:**
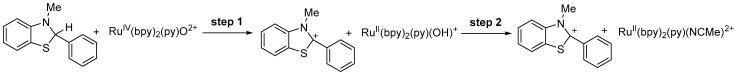
Proposed pathway of the reaction between [Ru**^IV^**O]**^2^**^+^ and **1H** in acetonitrile.

The first step is to acquire the ^1^H-NMR spectra of the key compounds. For the **^1^**H-NMR analysis, **1H** (R=H) (Equation (4)) and [Ru**^IV^**O]**^2^**^+^ (Equation (1)) were dissolved in deuteroacetonitrile (Figure 1). After 30 min of the dissolution process, the methyl peak (δ = 2.6 ppm) in **1H** (R=H) was almost three times higher than that in cation: 1^+^ (δ = 4.2 ppm), indicating that the reaction metering ratio between [Ru**^IV^**O]**^2^**^+^ and **1H**(R=H) was 1:1. Additionally, the characteristic signal δ = 9.4 ppm [20] indicated that the final Ru^II^ structure was Ru^II^(bpy)_2_(py)(NCCD_3_)^2+^. The reaction process is shown in Scheme 4.

**Figure 2:**
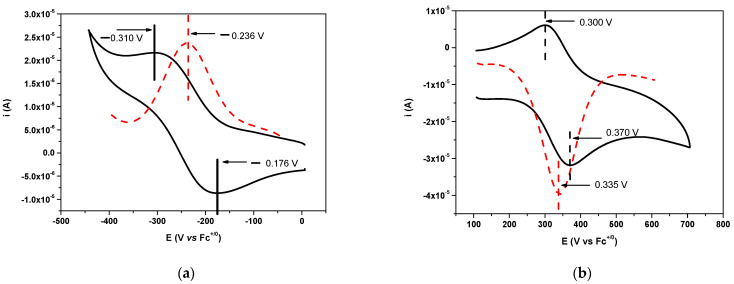
Cyclic voltammetry (CV) and Osteryoung square-wave voltammetry (OSWV) results of (**a**) Ru^IV^(bpy)_2_(py)(O)^2+^ and (**b**) Ru^II^(bpy)_2_(py)(OH)^+^ in anhydrous deaerated acetonitrile solution containing 0.1 M (n-Bu)_4_NPF_6_ as the supporting electrolyte: CV graph (black line), OSWV graph (red line). The sweep rate is 100 mV/s.

The second step is to obtain the thermodynamic data. The commonly used electrochemical measurement techniques include Cyclic voltammetry (CV) and Osteryoung square-wave voltammetry (OSWV). Herein, the single-electron potentials of two forms of Ruthenium-oxo-polypyridyl complexes ([Ru^IV^O]^2+^ and [Ru^II^OH]^+^ in Scheme 1) were determined using these approaches (Figure 2).

The obtained data are listed in Table 1. If the CV peak is flat (Figure 2a), the difference between the CV approach average and OSWV approach is large (Figure 2b). The CV approach is used only to indicate the reversibility of the compounds. Thus, these calculated values were derived from the data obtained using the OSWV approach.

∆*H*r×n is the molar enthalpy change of the reaction (Equation (2)) in acetonitrile at 298 K, which be calculated using titration calorimetry. In our previous study [21], we directly determined the molar enthalpy change of the reaction between benzothiazole iodized salt 1^+^ and a suitable organic hydride donor, 1,3-dimethyl-1,3-dihydro-2-phenyl-2H-benzimidazole, using isothermal titration calorimetry. Subsequently, we calibrated the hydride affinity of 1^+^: ∆*H* (1^+^I^−^) for 73.0kcal/mol, which is equal to the exact opposite of the C–H bond heterocleavage energy Δ*H*_H–D_(1H). The molar enthalpy change (Δ*H*r×n) of the reaction between the anions [Ru^IV^O]^2+^ and **1H** (Supporting Imformation) was determined; the relevant data are listed in Table 1. In terms of Equations (6)–(8), we also obtained the thermodynamic data for [Ru^IV^O]^2+^, which are also listed in Table 2.

**Figure 3:**
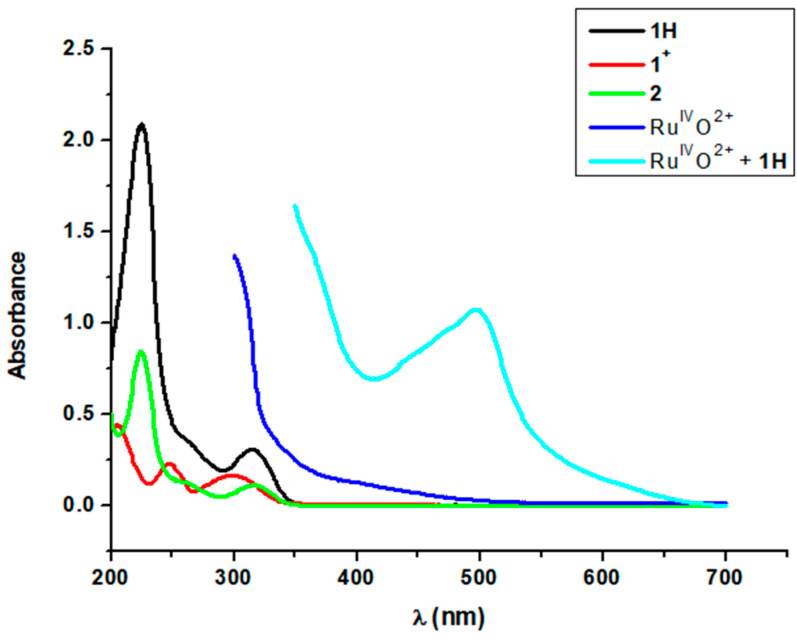
Absorbance spectra of the reactants and corresponding products in acetonitrile. The concentration of all the compounds is 0.1 mM/L. The reaction stoichiometry of **1H**:[Ru^IV^O]^2+^ is 1:1.

The third step is the collection of kinetic data. We dissolved equal amounts of [Ru^IV^O]^2+^ and **1H**(R=H), 1^+^, and 2 in acetonitrile to acquire their ultraviolet spectra in MeCN (Figure 3). Subsequently, the kinetic instrument (SX20 stopped-flow spectrometer) was used to detect the reaction of [Ru^IV^O]^2+^ with **1H**(R=H). The obtained kinetic curve exhibited a good pseudo-first-order correlation (Supporting Imformation). Moreover, when the concentration of **1H** (or **1D**) was altered, the rate constant *k*_obs_of these reactions showed a good linear correlation with the corresponding concentration of **1H** (or **1D**) (Supporting Imformation), indicating that the redox reaction for [Ru^IV^O]^2+^ with **1H** (or **1D**) was a good second-order reaction in the initial stage, which indicated a first-order correlation for both reactants. This is consistent with Dr. Yang Jin-Dong**’**s earlier findings based on hydride transfer reactions with high quantities of hydride compounds [22]. In addition, an isotope effect, KIE = *k*_1*H*_*/k*_1*D*_ ≈ 6, was obtained.

### 2.2. Results for the Reaction of Ruthenium-oxo-polypyridyl Compounds and ***2***

**Figure 4:**
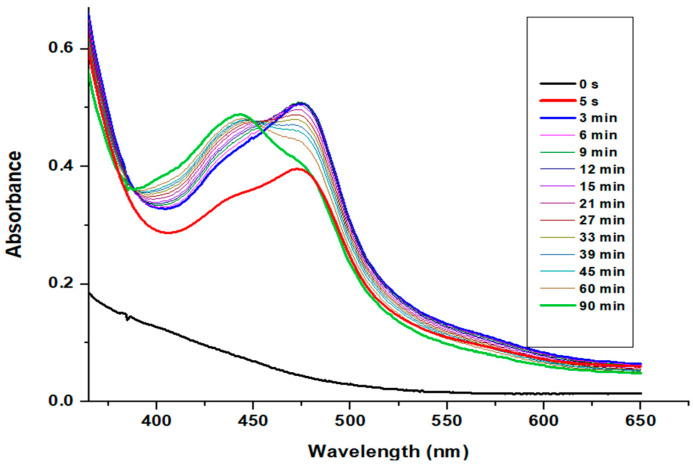
UV–visible spectral changes observed during the oxidation of **2** (1.25 mM) by [Ru**^IV^**O]**^2^**^+^ (6.26 × 10^−5^ M) in CH_3_CN: 0 s, 5 s, 3 min, 6 min, 9 min, 12 min, 15 min, 21 min, 27 min, 33 min, 39 min, 45 min, 60 min, and 90 min.

Variation in the ultraviolet spectrum of [Ru**^IV^**O]**^2^**^+^ reacting with **2** was observed. In acetonitrile, [Ru**^IV^**O]**^2^**^+^ was combined with 20 times the excess of **2**. The maximum absorption peak of Ru^II^(bpy)_2_(py)(NCCH_3_)^2+^ is λ = 445 nm [23], and the maximum absorption peak of the O-bound Ru^II^ intermediate is ~475 nm (Figure 4). As shown in Figure 4, a new absorption peak was formed at λ = 475 nm, which reached its maximum at 3 min and then gradually disappeared. This shows that once generated in acetonitrile, the O-bound intermediate was directly transformed into Ru^II^(bpy)_2_(py)(NCCH_3_)^2+^.

**Figure 5:**
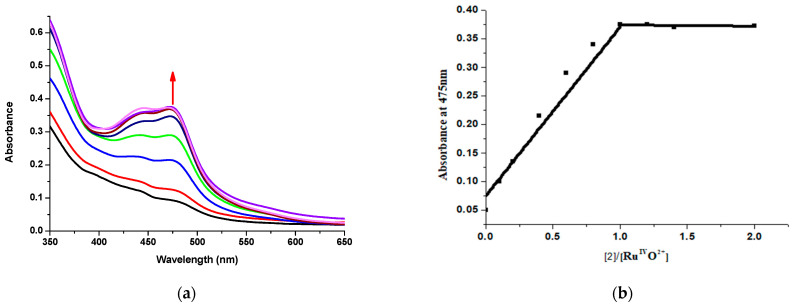
(**a**) Absorbance against wavelength 475 nm in the course of spectral titration. (**b**) Spectral titration to determine the stoichiometry of the reaction between **2** and [Ru**^IV^**O]**^2^**^+^ in acetonitrile.

Subsequently, we performed a titration experiment (Figure 5a), which further revealed that the stoichiometric ratio of the reaction was 1:1 (Figure 5b).

The ^1^H-NMR experiment was conducted after mixing a 1 mL CD_3_CN solution with 0.01 M (7.07 mg) [Ru**^IV^**O]**^2^**^+^ and 0.04 M (9.64 mg) of **2** for approximately 15 min.

### 2.3. Results for the Reaction of Ruthenium-oxo-polypyridyl Compounds and ***3H***

As the hydride affinity ∆*H*_H−D_ of **3H** (R=H) is relatively large (54.1 kcal/mol), we estimated that the reaction of **3H** with [Ru^IV^O]^2+^ would very likely be a one-step hydride transfer method. Following similar procedures, we synthesized **3H** (R=H, Cl, OCH_3_, NO_2_), and the reaction metering ratio between [Ru^IV^O]^2+^ and **3H**(R=H) proved to be 1:1. The kinetic isotope effect (KIE ≈ 3), meanwhile, the relationship between substituent and activation energy (linear correlation), was investigated. The thermodynamic parameters of the reactants and reaction intermediates for **3H** could still be detected on the basis of the electrochemical and calorimetric data. Thus, the thermodynamic platform of the reaction was established (Supporting Imformation).

## 3. Discussion

### 3.1. Considerations Regarding the Reaction of Ruthenium-oxo-polypyridyl Compounds and ***1H***

**Scheme 5:**
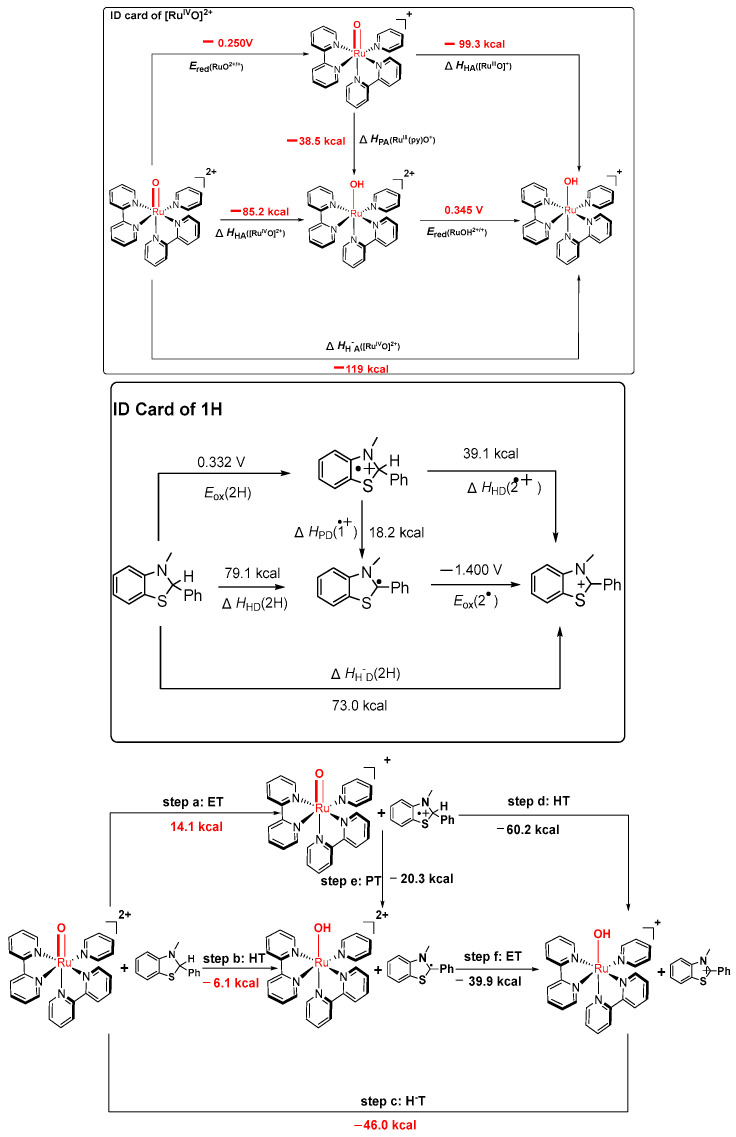
Thermodynamic analytic platform for the mechanism of hydride transfer from **1H**(R=H) to [Ru^IV^O]^2+^ at 298 K in acetonitrile.

Based on the preceding thermodynamic data, the molecular identity (molecular ID) of the two compounds was determined (Scheme 5). Although the change in state energy was negative for both hydrogen and hydride, the change in the state energy of hydride was substantially smaller than the change in state energy produced by hydrogen during the reaction of [Ru^IV^O]^2+^ with **1H**(R=H). This indicates that the mechanism of the preceding reaction leans toward a thermodynamic one-step hydride transfer.

**Figure 6:**
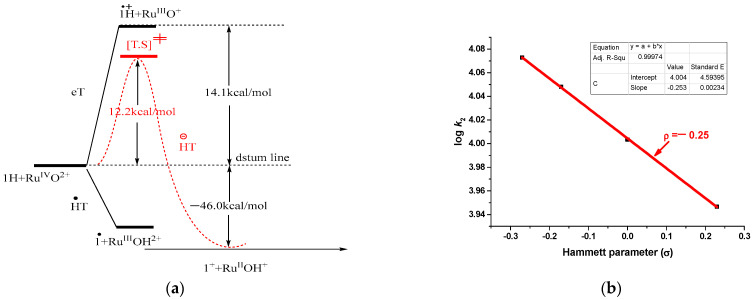
(**a**) Comparison of changes in energy state for the three possible initial steps of the hydride transfer from **1H**(R=H) to [Ru^IV^O]^2+^ and the activation free energy of the hydride transfer. (**b**) Hammett plot of log *k*_2_ for the hydride transfer from **1H** to [Ru^IV^O]^2+^ vs. σ at 298 K in MeCN under anaerobic conditions.

From a different perspective, the Gibbs free energy of the elementary reaction must be larger than or equal to the change in state energy of any elementary step. The energy of electron transfer, as the initial step (14.1 kcal/mol), exceeds the Gibbs free energy change of 12.2 kcal/mol in the corresponding reaction, while the energy change for hydrogen transfer, as the initial step, is −6.1 kcal/mol. The energy change of −46.0 kcal/mol for one-step hydride transfer is smaller than this value, indicating that taking the mechanism of electron transfer as the key step is illogical (Figure 6a). As stated previously, [Ru^IV^O]^2+^ has a large isotope impact with **1H**(R=H) and **1D** reactions, *k*_1H_*/k*_1*D*_ = 6, showing that the rate-determining step of a chemical reaction causes the disruption of chemical bonds, which is not electron transfer but hydride transfer.

The index of the second-order rate constant log *k*_2_ for the reaction of 2-substituted 2,3-dihydrobenzothiazolines with [Ru^IV^O]^2+^ demonstrates a normative linear correlation with the Hammett constant of its substituent (Figure 6b), with its slope, ρ < 0, indicating partial positive charge accumulation in the reaction center during the transition state. This also enables the one-step hydride transfer method to take place.

### 3.2. Considerations Regarding the Reaction of Ruthenium-oxo-polypyridyl Compounds and ***2***

**Figure 7:**
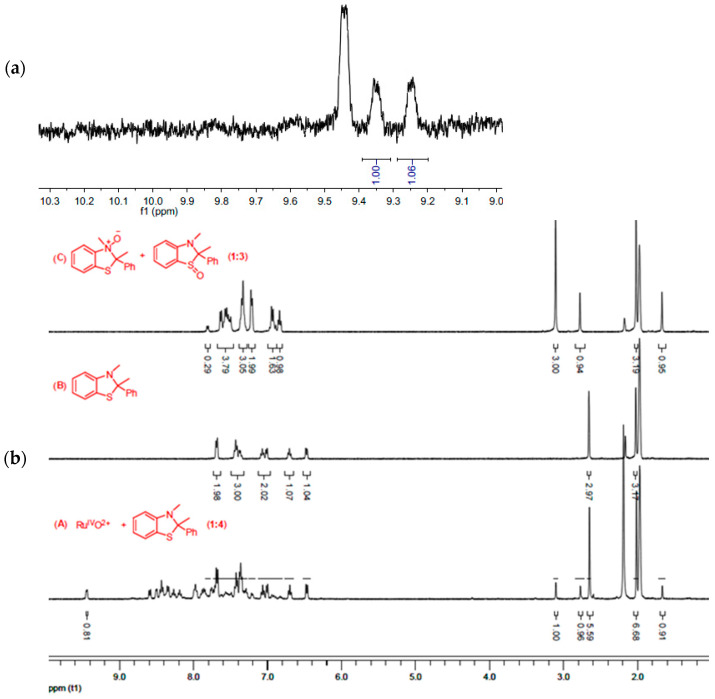
(**a**) Partial ^1^H-NMR spectra of a reaction mixture in CD_3_CN containing [Ru^IV^O]^2+^ (0.01 M) with **2** (0.04 M) after 15 min. (**b**) Overlap in the ^1^H-NMR spectra of (A) a reaction mixture in CD_3_CN containing [Ru^IV^O]^2+^ (0.01 M) with **2** (0.04 M) after 12 h, (B) **2** in CD_3_CN, and (C) a mixture of 2,3-dimethyl-2-phenyl-2,3-dihydrobenzo[d]thiazole 1-oxide and 2,3-dimethyl-2-phenyl-2,3-dihydrobenzo[d]thiazole 3-oxide in CD_3_CN.

δ = 9.44 ppm represents the typical signal of Ru^II^(bpy)_2_(py) (NCCD_3_)^2+^ [3], whereas δ = 9.35 ppm and δ = 9.25 ppm (Figure 7a) represent the O-bound intermediates in **2**, in which the relevant sulfur and nitrogen atoms are oxidized. ^1^H-NMR studies of the reaction mixture (Figure 7b) performed 12 h later revealed that the δ = 9.35 ppm and δ = 9.25 ppm signals of the intermediates in the low-field area had disappeared, leaving only δ = 9.44 ppm. This demonstrated that all Ru^II^ intermediates were transformed into Ru^II^(bpy)_2_(py)(NCCD_3_)^2+^.

2,3-Dimethyl-2-phenyl-2,3-dihydrobenzo[d]-thiazole-1-oxide (S-oxide) and 2,3-dimethyl-2- phenyl-2,3-dihydrobenzo[d]thiazole-3-oxide (N-oxide) were also synthesized based on studies conducted previously [24]. Since S-oxide and N-oxide are very unstable and difficult to separate and purify, the above signals (δ = 9.35 ppm and δ = 9.25 ppm) are only reasonable estimations. A comparison of their ^1^H-NMR spectra for the reaction mixture revealed that the oxide product of **2** comprised both S-oxide and N-oxide. Their ratio generated in the reaction was 3:1. Based on the methyl peak integral ratio of the intermediate and the methyl peak integral ratio of the product after 12 h, the final ratio of S-oxide to N-oxide was 1:1. After 12 h, the ^1^H-NMR spectra indicated that the ratio of [Ru^IV^O]^2+^ to **2** consumed in the process was 1:1. When N or S atoms in **2** are oxidized to N-oxide or S-oxide, it is believed that electron absorption by the neighboring N-oxide or S-oxide will lower the electron cloud density on the other S or N atom in **2**.

**Scheme 6:**
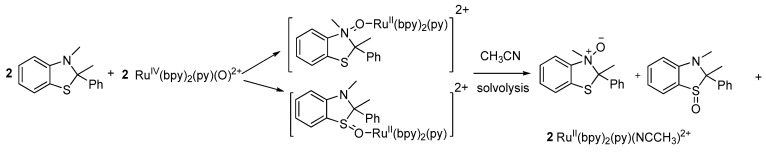
Reaction pathway of [Ru^IV^O]^2+^ and 2 in acetonitrile.

From the above experimental phenomenon, we can conclude that Scheme 6 describes the detailed path of the oxidation reaction, in which the oxygen atoms in [Ru^IV^O]^2+^ combine with N or S atoms in **2** to form two O-bound intermediates, which are directly dissolved into Ru^II^(NCCD_3_)^2+^ and equal amounts of S-oxide and N-oxide.

Because the steric hindrance difference in the reaction may be disregarded, the second-order rate constant *k*_2_ for the [Ru^IV^O]^2+^ oxidation of **1H** ranges from 10^3^ to 10^4^ M^−1^s^−1^, which is approximately three orders greater than the second-order rate constant *k*_2_ (9.59 M^−1^s^−1^) for the [Ru^IV^O]^2+^ oxidation of **2** (Supporting Imformation). Therefore, we deduce that the kinetics govern the selectivity of C–H bonds at position 2 in [Ru^IV^O]^2+^ oxidation for **1H**.

### 3.3. Considerations Regarding the Reaction of Ruthenium-oxo-polypyridyl Compounds and ***3H***

The kinetic isotope effect of the reaction of [Ru^IV^O]^2+^ with **3H** (*k*_3H_/*k*_3D_ = 3) and the relationship between the substituents’ constant and activation energies can negate the hydrogen atom transfer mechanism. As the enthalpy change for hydride transfer is much smaller than the enthalpy change for electron transfer, the thermodynamic analytic platform (Supporting Imformations) can also prove that the reaction mechanism between them is a one-step hydride transfer method. We deem that when the difference in the enthalpy change is large enough (greater than 50 kcal/mol), the chemical reaction will be likely to proceed with a one-step hydride transfer mechanism.

### 3.4. Considerations Regarding the Novelty of This Work

First, a new type of reaction was discovered. The reaction mechanism of a metal–organic complex with an organic hydride donor was studied. The one-step hydride transfer method was validated.

**Scheme 7:**
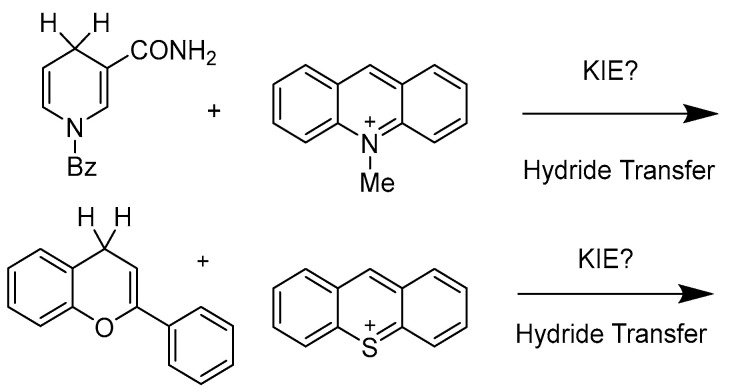
The KIE are not available.

Second, the advantages of a new mechanism derivation approach are presented. For the DFT method, the appropriate unit and functional and dispersion correction should be applied to this system; otherwise, the numerical deviation will be significant. However, the suitable functional and the unit for Ruthenium-oxo-polypyridyl compounds were not reported. In this case, the workload is larger, and the time is long. For the isotopic effects method, the deuterium compounds of many compounds cannot be synthesized. There are many compounds whose their isotopic effects cannot be obtained: when two C–H bonds are present at the same active site, we cannot hold that one of the C–H bonds is unchanged while, simultaneously, the other C–D bond is completely separated in the hydride transfer reaction (Scheme 7). Therefore, we can omit the DFT method and the isotope effect method and infer the reaction mechanism based on the method of thermodynamics combined with kinetics.

Third, a new mechanism derivation approach is presented. The point of using the kinetic method is essentially to prove that the dependence of the substituent constant and activation energy works to negate the hydrogen atom transfer path. Based on the reactions of compounds **1H** and **2** with [Ru^IV^O]^2+^, it is indicated that the difference in the reaction rate between the two paths leads to the selectivity of the reaction, although the magnitude of the rate difference that can determine the selectivity of the reaction path is not very precise. Investigating thermodynamic methods, the difference in enthalpy change between the two paths leads to selectivity, although the enthalpy change difference that determines the selectivity of the path is still not very precise. We studied the reactions of compounds **1H** and **3H** with [Ru^IV^O]^2+^, determining that the difference in enthalpy change should be less than 50 kcal/mol.

## 4. Conclusions

First, through ^1^H-NMR analysis and spectral titration, as well as thermodynamic and kinetic methods, the reaction mechanism between [Ru^IV^O]^2+^ and **1H**, **3H** was confirmed to be a one-step hydride transfer. As [Ru^IV^O]^2+^ reacts with **2** in a ratio of 1:1, **2** produces two intermediates with a stoichiometric ratio of 1:1, which are dissolved directly into Ru^II^(NCCD_3_)^2+^, along with equal amounts of S-oxide and N-oxide.

Second, the advantage of the new mechanism method lies in its capacity to remedy the limitation of the DFT calculation method for kinetic isotope effect diagnosis. Its essence lies in the study of the selectivity of reaction enthalpy change and rate constant difference with respect to the reaction path, and the judgment of the substituent effect on the hydrogen atom transfer path. Third, the difference in the reaction rate and enthalpy change between the two paths both leads to the selectivity of the reaction.

## Figures and Tables

**Table 1:** Reaction heat (∆*H*r) of Ru^IV^(bpy)_2_(py)(O)^2+^ with **1H**, hydride affinities of Ru^IV^(bpy)_2_(py)(O)^2+^, and redox potentials of relative species in acetonitrile.

[Ru^IV^O]^2+^	∆*H*_r_^a^	∆*H*_H−A_[Ru^IV^O]^2+ b^	*E*^°^_red_(RuO^2+/+^) ^c^		*E*^°^_ox_(RuOH^+/2+^) ^c^	
			CV	OSWV	CV	OSWV
	−64.9	−119	−0.243	−0.250	0.335	0.335

^a^ ∆*H*r was obtained from the reaction heat using Equation (2). ^b^ ∆*H*_H−A_ was obtained from reference [21]. ^c^ Measured via CV and OSWV methods in acetonitrile at 298 K. The units are in volts vs. Fc^+^/Fc^0^ and are reproducible to 5 mV or greater.

**Table 2:** Enthalpy changes of Ru^IV^(bpy)_2_(py)(O)^2+^ to obtain a hydrogen atom and enthalpy changes of Ru^II^(bpy)_2_(py)(OH)^+^ to obtain a proton and a neutral hydrogen atom in acetonitrile (kcalmol^−1^) ^a^.

[Ru^IV^O]^2+^	∆*H*_HA_[Ru^IV^O]^2+ b^	∆*H*_PA_[Ru^III^O]^2+ b^	∆*H*_HA_[Ru^III^O]^2+ b^
H	−85.2	−38.5	−99.3

^a^ Relative uncertainties were estimated to be smaller than or close to 1 kcal/mol in each case. ^b^ Because the values obtained via OSWV are closer to the corresponding standard redox potentials than the values obtained via CV herein, taking the values *E*°(H^+/0^) = −2.307 (V vs. Fc^+/0^) and *E*°(H^0/−^) = −1.137 V (V vs. Fc^+/0^) (Fc = ferrocene) and the redox potentials of [Ru^IV^O]^2+^ and [Ru^II^OH]^+^, measured using the OSWV method (Table 1), *E*°(RuO^2+/+^) and *E*°(RuOH^+/2+^) were selected.

## Data Availability

Not applicable.

## Supplementary Materials

The following supporting information can be downloaded at: https://www.mdpi.com/article/10.3390/molecules28114401/s1. Detailed ^1^H-NMR and ^13^C-NMR data of the typical compounds are shown in the SI. Refs [25,26,27,28,29,30,31] are cited in the Supplementary Materials.
