# Supplementary material for: A Mechanism Study of Redox Reactions of the Ruthenium-oxo-polypyridyl Complex
Source: Molecules. 2023 May 28;28(11):4401. doi: 10.3390/molecules28114401 (PMC10254183; doi:10.3390/molecules28114401)
Supplement: Supplementary file 1 [file molecules-28-04401-s001.zip › molecules-2393577-supplementary.pdf]

Supporting Information for

A Mechanism Study of Redox Reactions of the Ruthenium-oxo-polypyridyl Complex

Bao-Long Chen, Sheng-Yi Yan, and Xiao-Qing Zhu
The State Key Laboratory of Elemento-Organic Chemistry, Collaborative Innovation Center of Chemical Science and Engineering, College of Chemistry, Nankai University, Tianjin 300071, P. R. China.
Correspondence: cblnku@163.com (B.-L.C.); xqzhu@nankai.edu.cn (X.-Q.Z.)

Table of Contents

**SI.**	**Materials and syntheses.**	**S1**
**SII.**	**Measurement of redox potentials.**	**S3**
**SIII.**	**Isothermal titration calorimetry (ITC).**	**S3**
**SIV.**	**^{1}H-NMR and ^{13}C-NMR spectra of representative compounds**	**S4-S13**
**SV.**	**Kinetic analysis of reactions for $[Ru^{IV}O]^{2+}$**	**S14**
**SVI.**	**Thermodynamic analysis of reactions for $[Ru^{IV}O]^{2+}$ with 3H**	**S15**

SI. Materials and syntheses.

Solvents and reagents were obtained from commercial sources and used as received unless otherwise noted. Reagent-grade acetonitrile was refluxed over $KMnO_4$ and K_2CO_3 for at least eight hours and doubly distilled over P_2O_5 under argon and stored in a glove box before use. All compounds were synthesized (Figure S1) according to the literature methods ($[Ru^{IV}O]^{2+}$[1-4],1H[5], 2[6,7], 3H[5]).

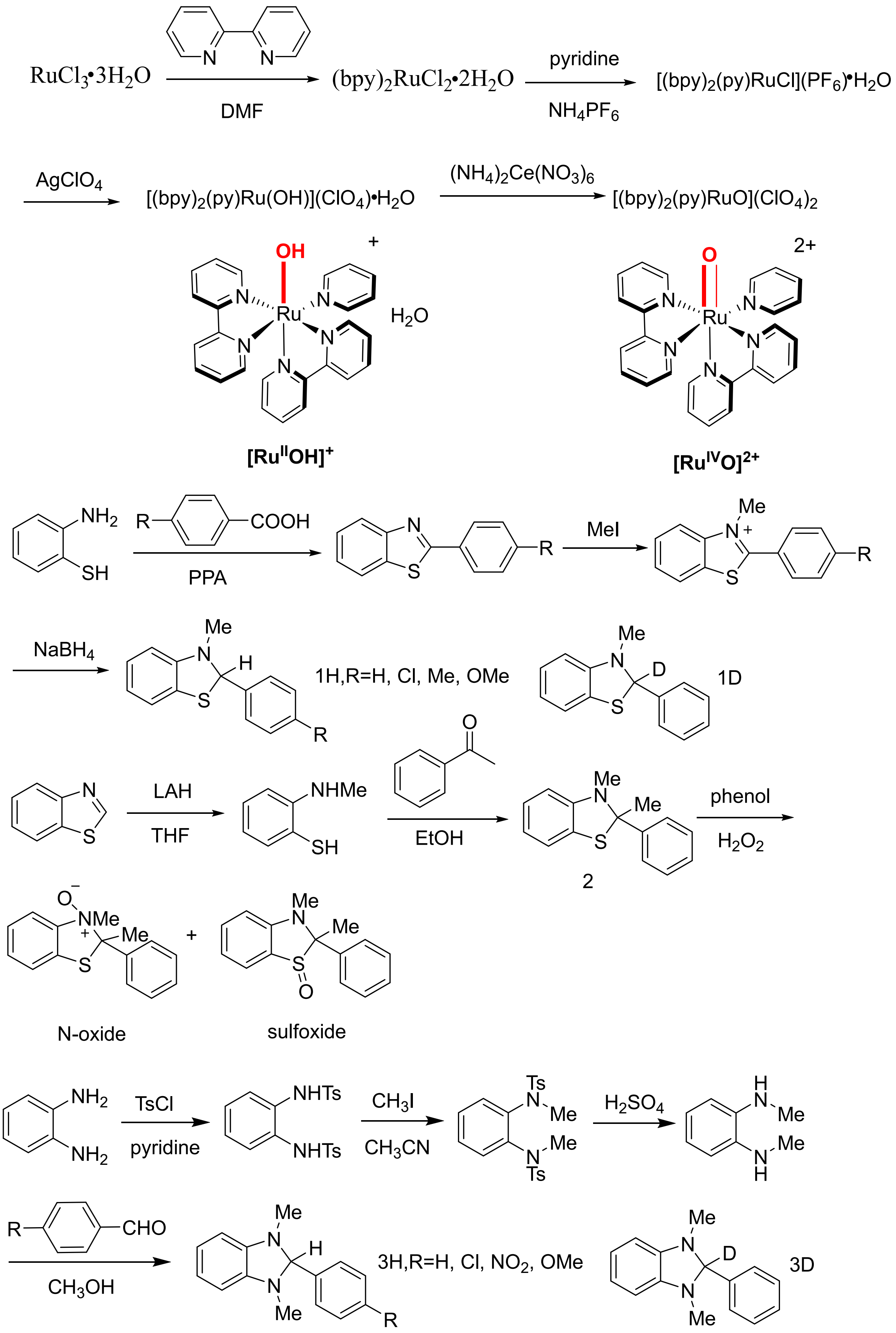

Figure S1. The synthesis route for all compounds.

SII. Measurement of redox potentials.

The electrochemical experiments were carried out via cyclic voltammetry (CV) and Osteryoung square-wave voltammetry (OSWV)[8] using a BAS100B electrochemical apparatus in a de-aerated acetonitrile under argon atmosphere at 298 K, as described previously. nBu_4NPF_6 (0.1M) in acetonitrile was employed as the supporting electrolyte. The standard three-electrode cell consisted of a glassy carbon disk as a working electrode, a platinum wire as a counter electrode, and 0.1 M $AgNO_3/Ag$ (in 0.1 M n-Bu_4NPF_6- acetonitrile) as a reference electrode. The ferrocenium/ferrocene redox couple ($Fc^{+/0}$) was taken as the internal standard. The reproducibility of the potentials was usually ≤5 mV for ionic species and ≤10 mV for neutral species.

SIII. Isothermal titration calorimetry (ITC).

The titration experiments were performed using a CSC4200 isothermal titration calorimeter in acetonitrile at 298 K, as described previously [9]. The performance of the calorimeter was checked by measuring the standard heat of neutralization of an aqueous solution of sodium hydroxide with a standard aqueous HCl solution. The solvents used in the experiment were anhydrous and anaerobic acetonitrile. $Ru^{IV}(bpy)_2(py)(O)^{2+}$ (1 mM) was used as the titration solution, and 1 mL carbonyl compound anion XH was used as the reaction solution. The experiment was determined at 298K, the balance time was 400 s, and the titration time was 400 s. The heat of the reaction was obtained by integrating the area of each peak (except for the first peak) in the titration curve. The test was repeated at least 5 times for each sample, and the reaction heat error was ≤±0.5 kcal/mol. Note: typically, the first injection shows less heat than expected. This is often due to diffusion across the tip of the needle or to difficulties in positioning the buret drive.

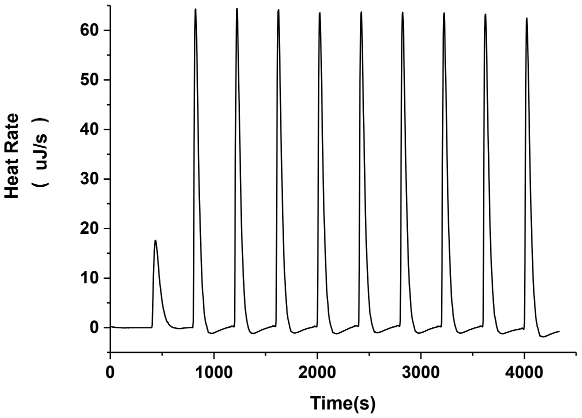

Figure S2. Isothermal titration calorimetry for determining the heat of the reaction between $[Ru^{IV}O]^{2+}$ and 1H (R = H) in acetonitrile at 298 K. The titration was conducted by adding 10 μL of $[Ru^{IV}O]^{2+}$ (1.1 mM) every 400 s to acetonitrile containing the 1H (R = H) (10 mM).

SIV. ¹H-NMR and ¹³C-NMR spectra of representative compounds.

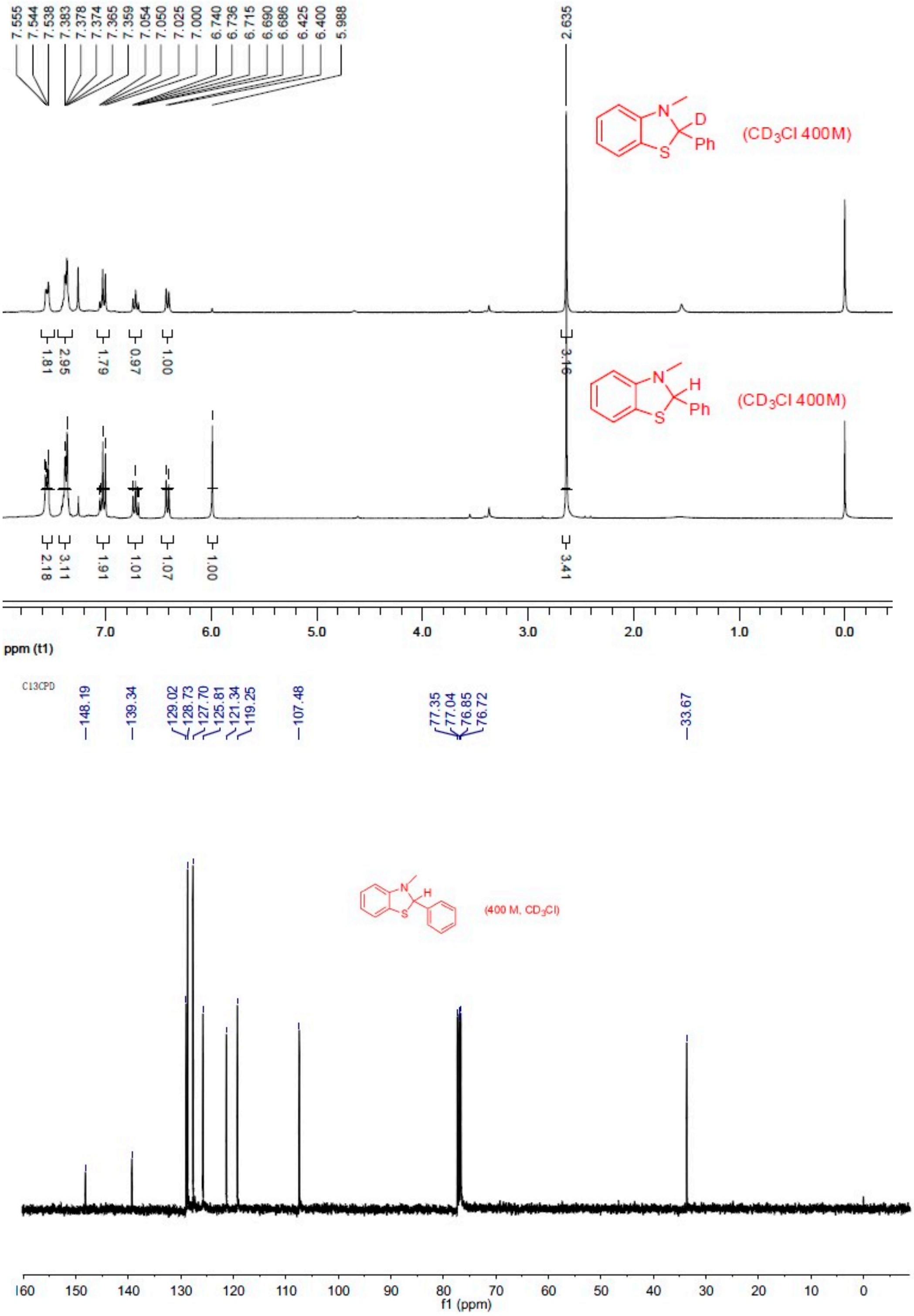

Scheme S1. ¹H-NMR spectra of 1H and 1D(R=H) and ¹³C-NMR spectra of 1H(R=H).

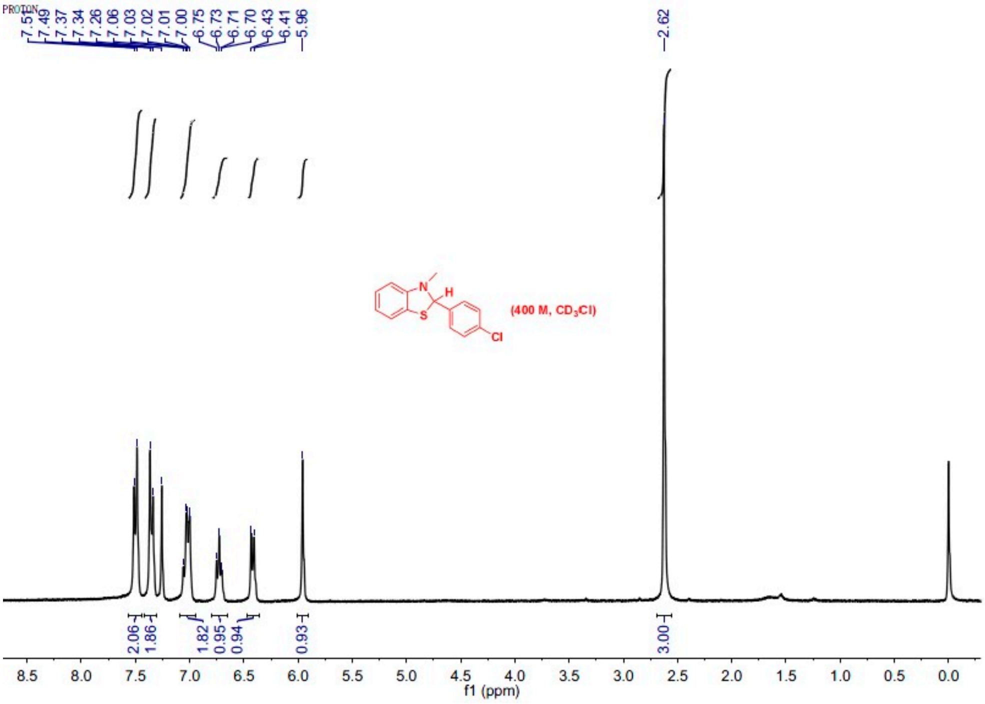

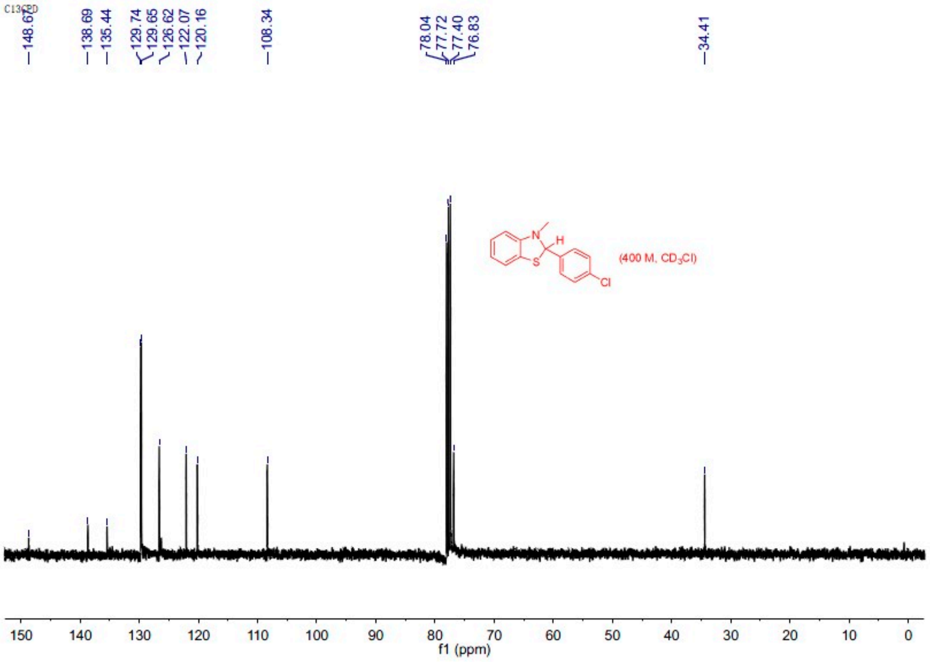

Scheme S2. ^{1}H-NMR and ^{13}C-NMR spectra of 1H(R=Cl).

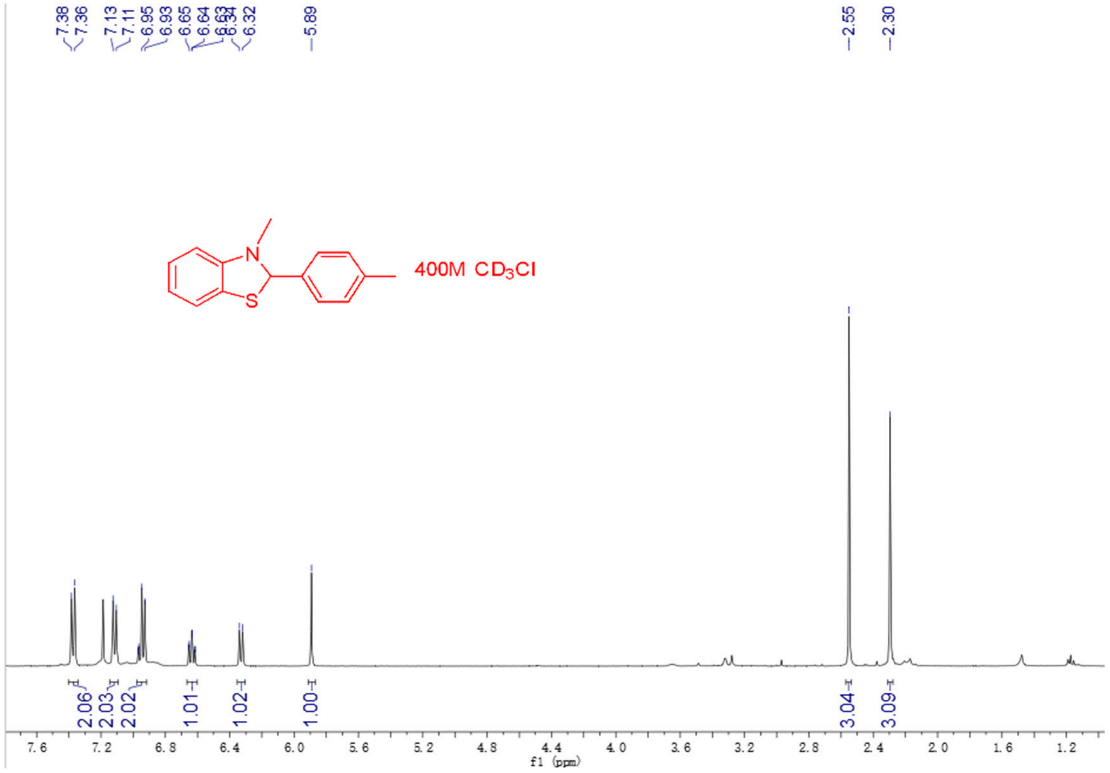

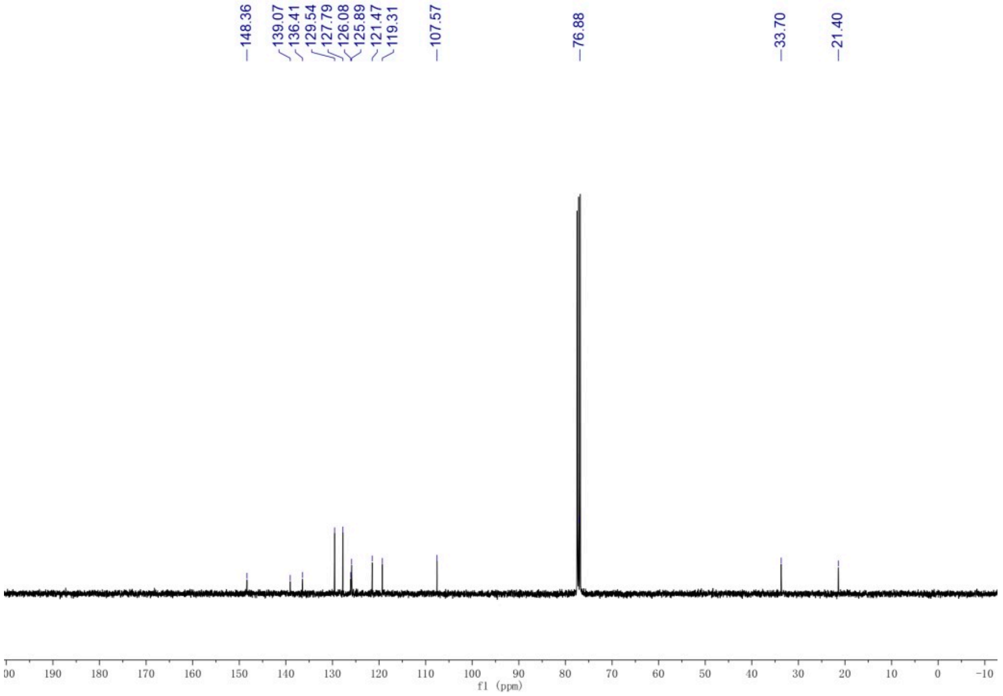

Scheme S3. ^{1}H-NMR and ^{13}C-NMR spectra of 1H(R=Me).

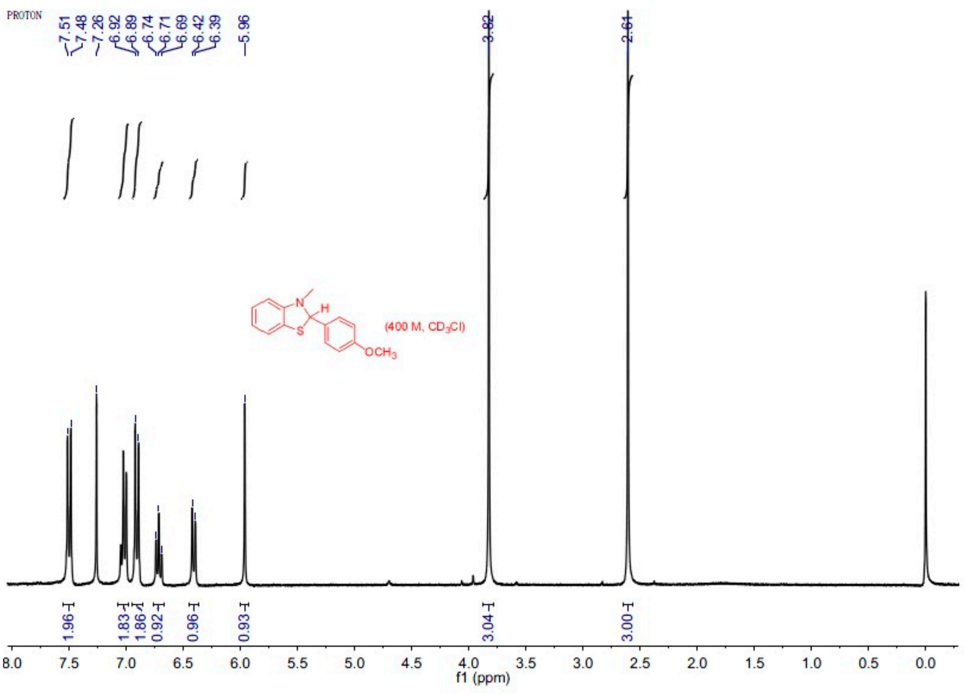

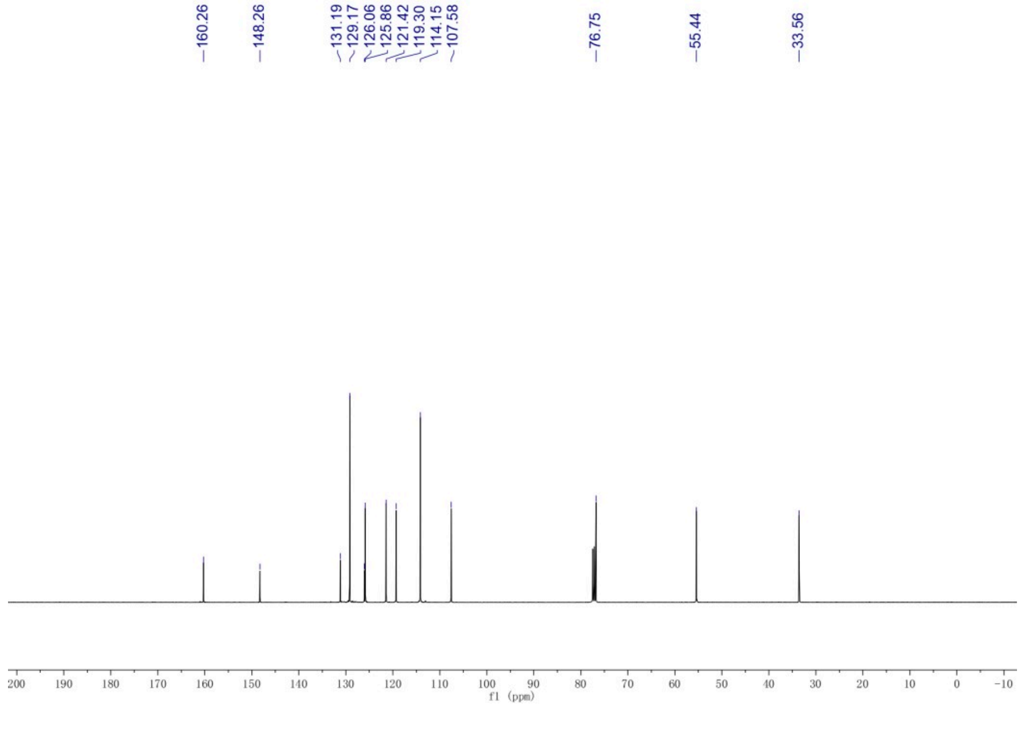

Scheme S4. ^{1}H-NMR and ^{13}C-NMR spectra of 1H(R=OMe).

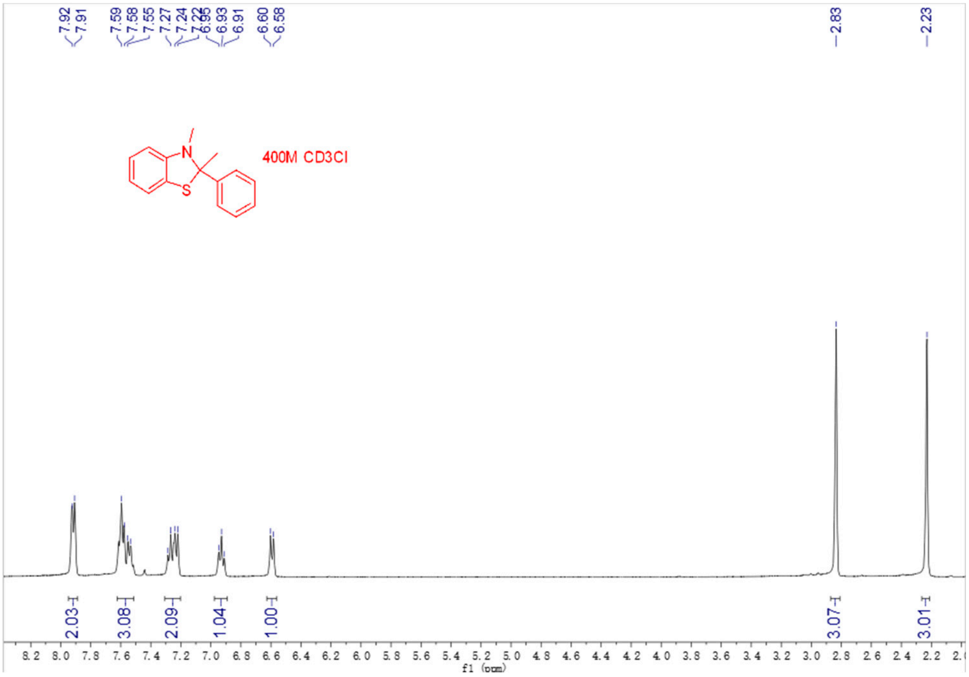

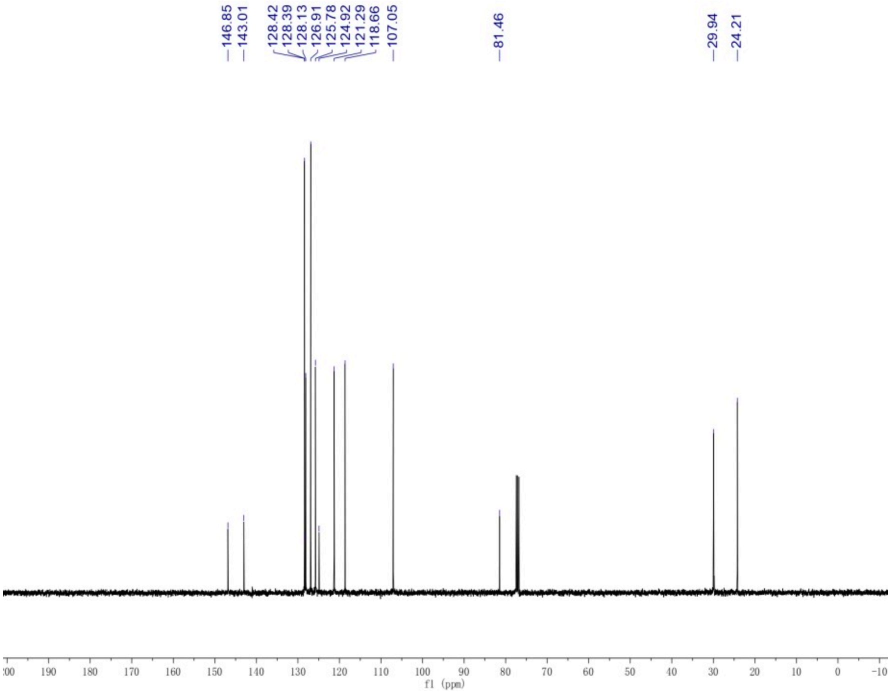

Scheme S5. ^{1}H-NMR and ^{13}C-NMR spectra of 2.

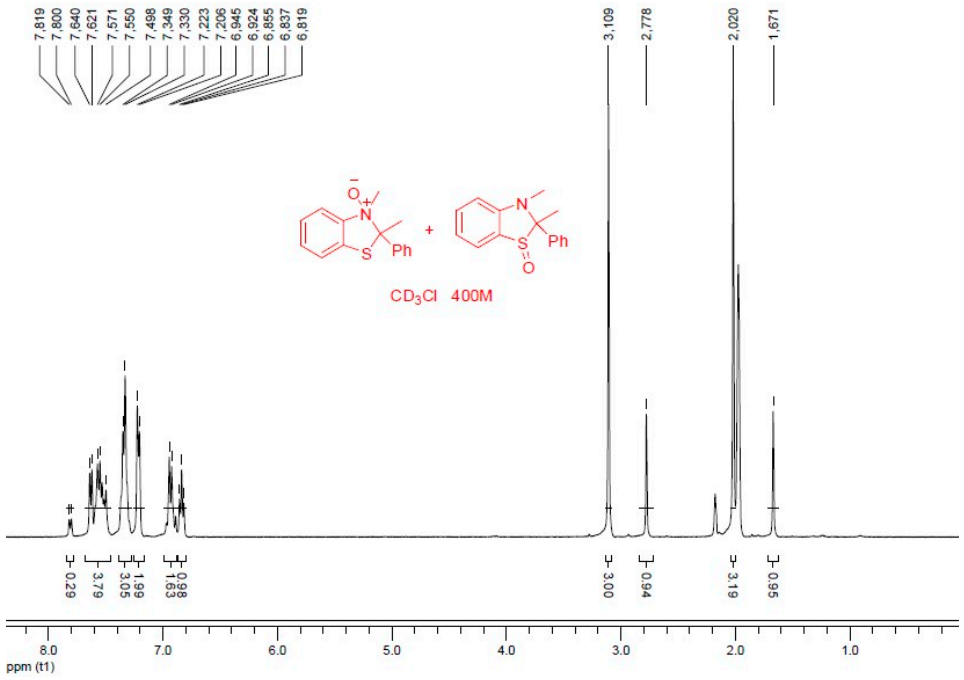

Scheme S6. ^{1}**H-NMR spectra of N-oxide and sulfoxide.**

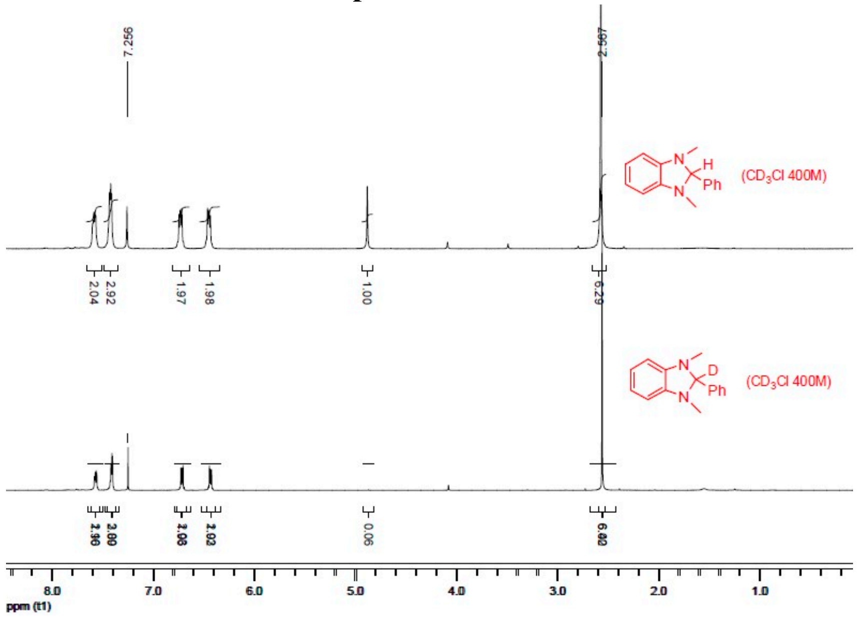

Scheme S7. ^{1}**H-NMR spectra of 3H and 3D.**

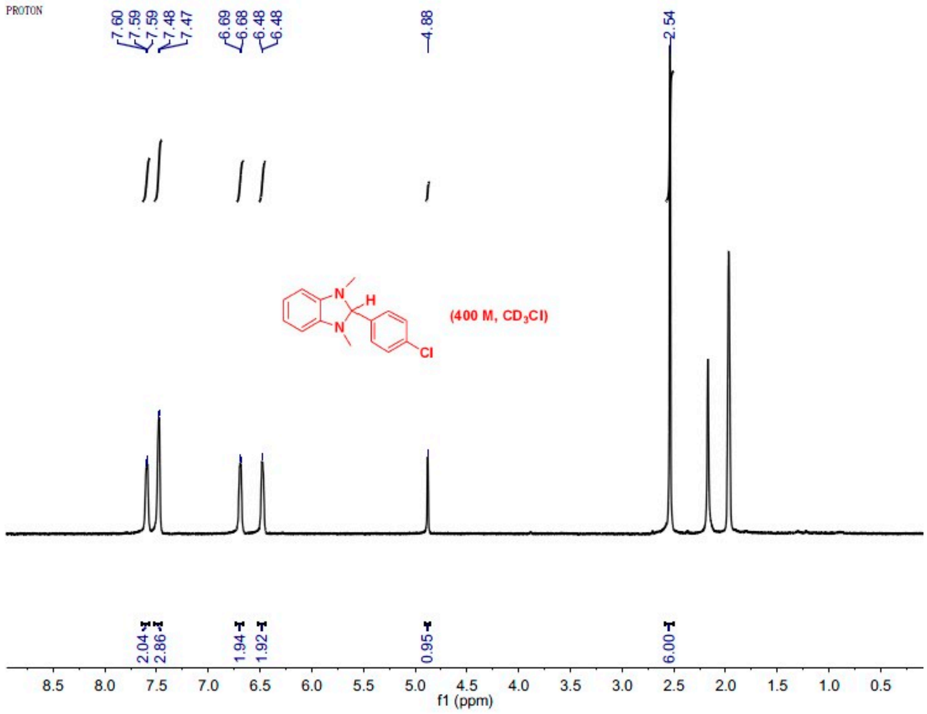

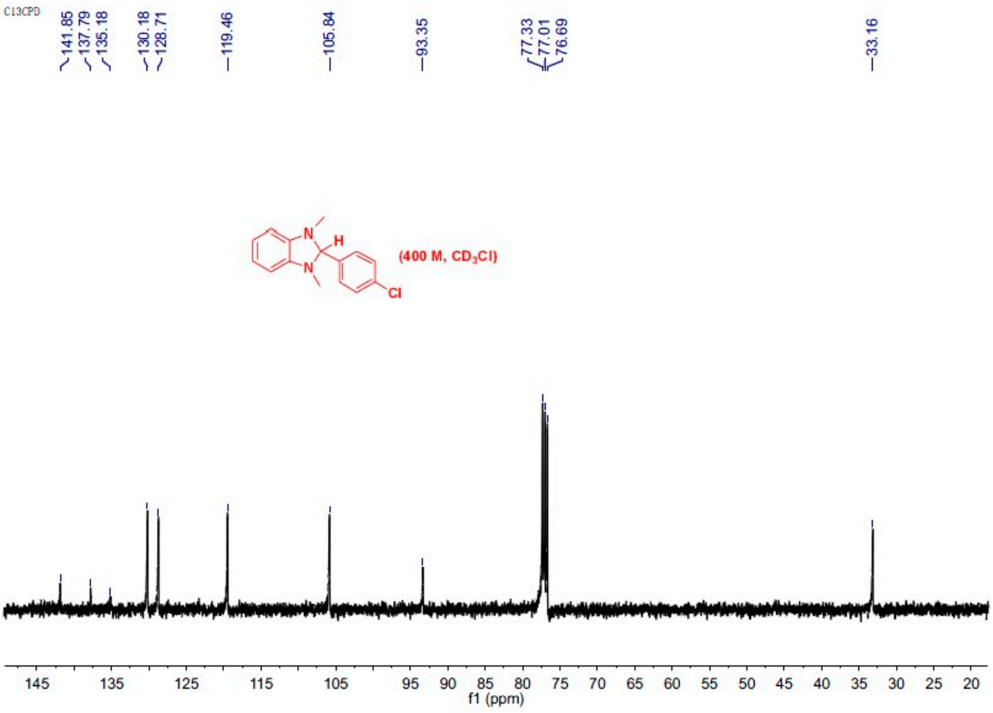

Scheme S8. ^{1}H-NMR and ^{13}C-NMR spectra of 3H （R=Cl）.

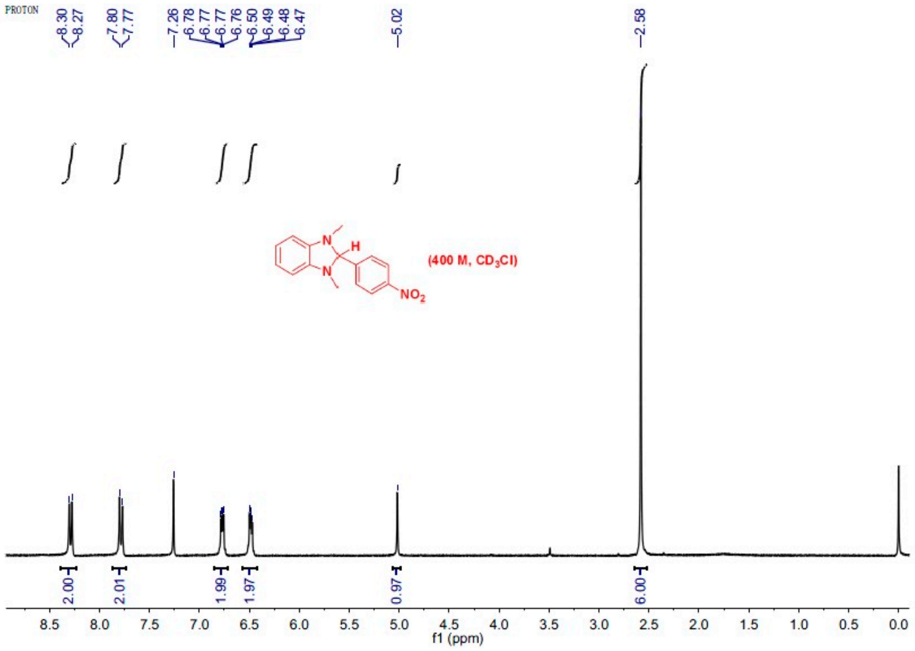

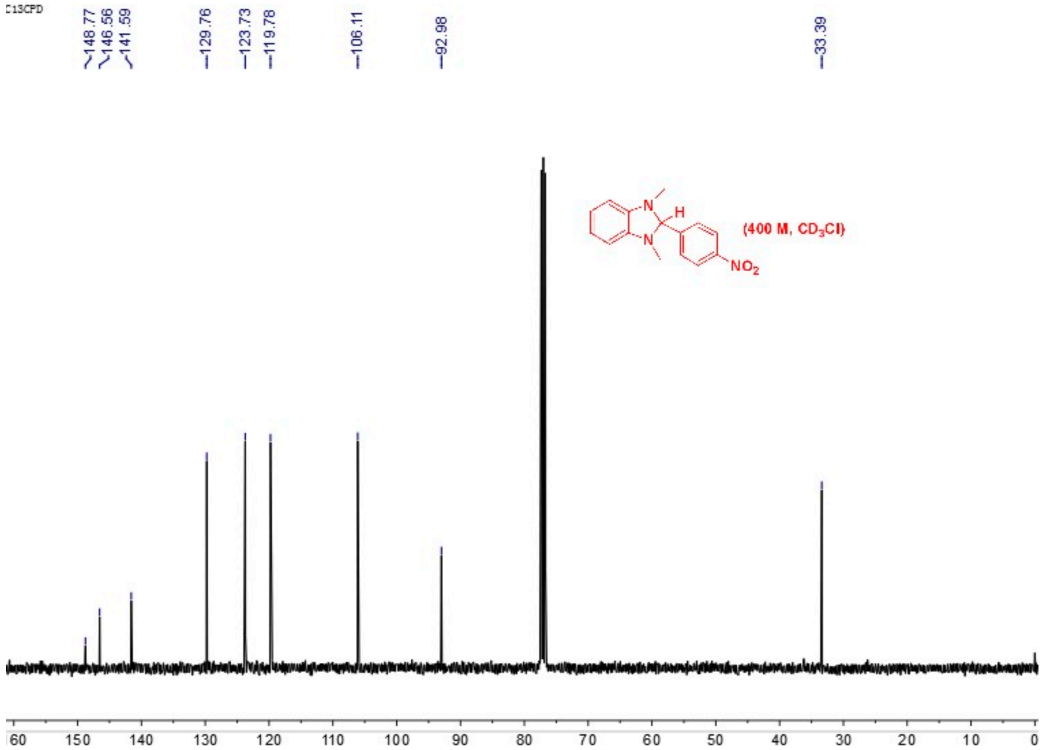

Scheme S9. ^{1}**H-NMR and** ^{13}**C-NMR spectra of 3H（R=NO_2）.**

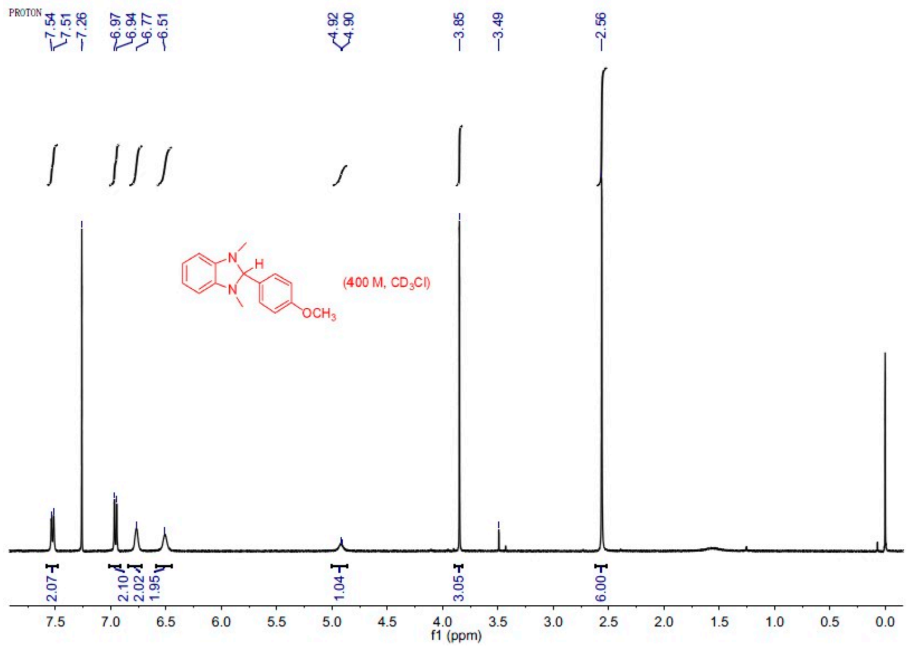

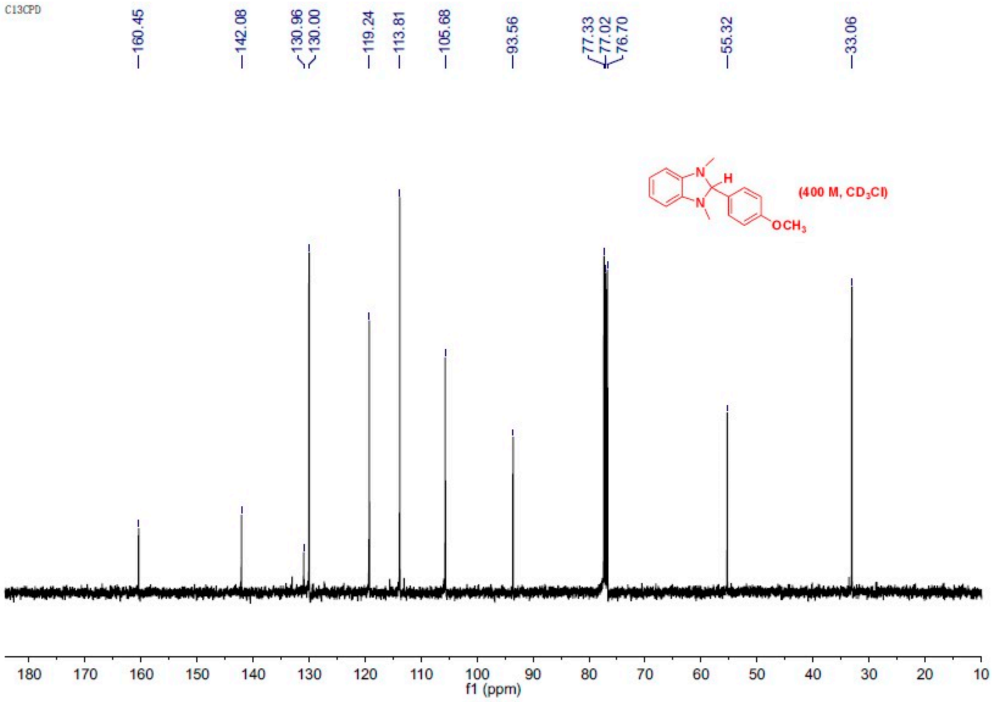

Scheme S10. ^{1}H-NMR and ^{13}C-NMR spectra of 3H（R=OCH_3）.

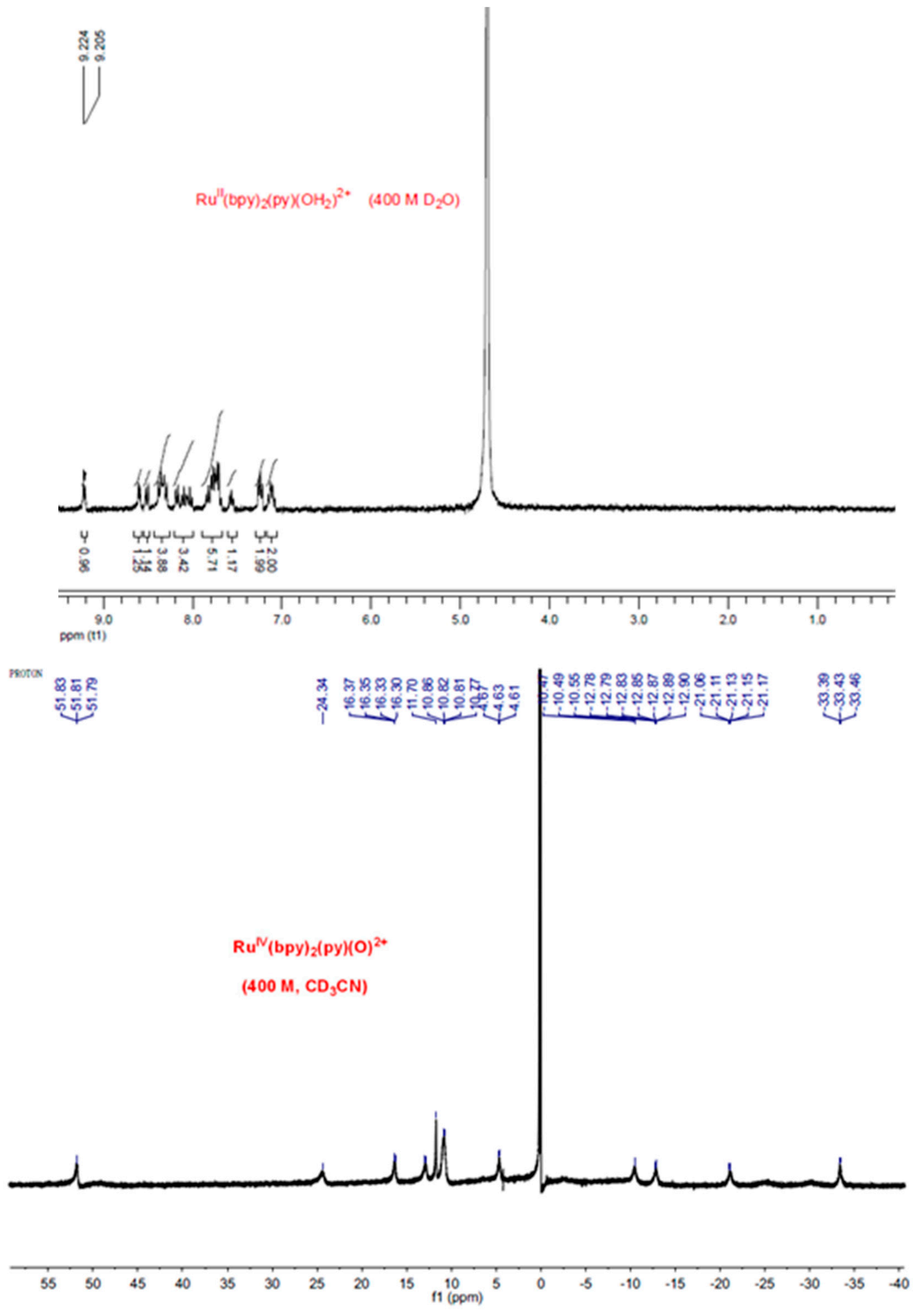

Scheme S11. ^{1}H-NMR spectra of [Ru^{II}（OH）]$^+$ and [Ru^{IV}O]$^{2+}$

SV. Kinetic analysis of reactions for $[Ru^{IV}O]^{2+}$.

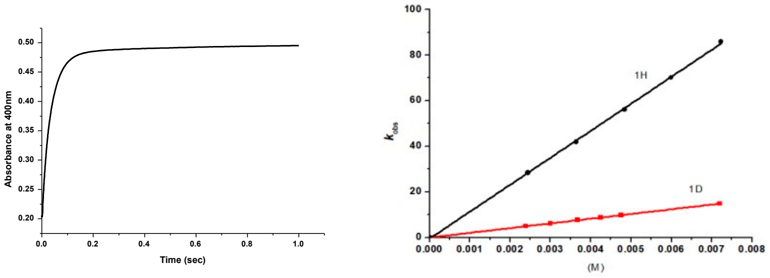

(a) (b)

Figure S3. Absorbance change at 497 nm in the initial phase of 1H (R = H) oxidation by $[Ru^{IV}O]^{2+}$ at 298K in MeCN under anaerobic conditions. $[1H]0 = 2.4 \times 10^{-3}$ M, $[Ru^{IV}O]^{2+}{}_0 = 1.2 \times 10^{-4}$ M. The solid line represents the first-order kinetics. (b) Pseudo-first-order plot for the kinetics of 1H (R = H) and 1D with $[Ru^{IV}O]^{2+}$ at 298K in MeCN under anaerobic conditions.

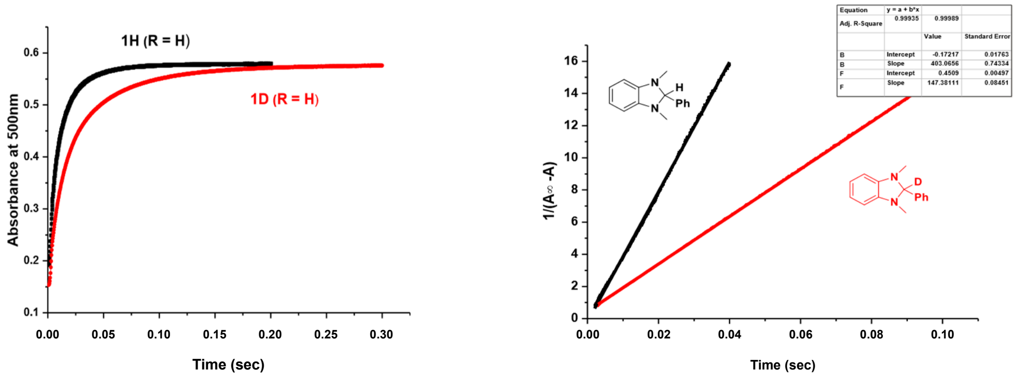

（a）

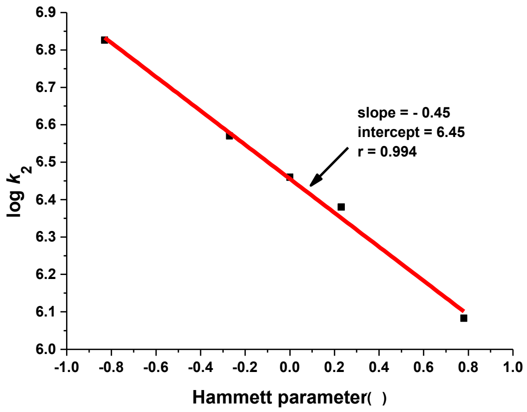

（b）

Figure S4. (a) Absorbance change and second-order plot at 500 nm in the initial phase of 3H (R = H), with 3D oxidation by $[Ru^{IV}O]^{2+}$ at 298K in MeCN under anaerobic conditions. $[1H]^0 = [[Ru^{IV}O]^{2+}]^0 = 1.8 \times 10^{-4}$ M.

（**b**）Hammett plot of logk_2 of the hydride transfer from 3H to $[Ru^{IV}O]^{2+}$ vs. σ at 298K in acetonitrile under anaerobic conditions.

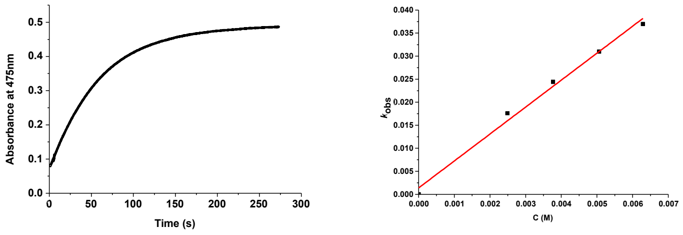

(a) (b)

Figure S5. (a) Absorbance change at 475 nm in the initial phase of 2 oxidation by $[Ru^{IV}O]^{2+}$ at 298 K in MeCN. (b) Plots of k_{obs} vs. the concentration of 2 in the oxidation of 2 by $[Ru^{IV}O]^{2+}$ in MeCN at 298 K.

SVI. Thermodynamic analysis of reactions for $[Ru^{IV}O]^{2+}$ with 3H.

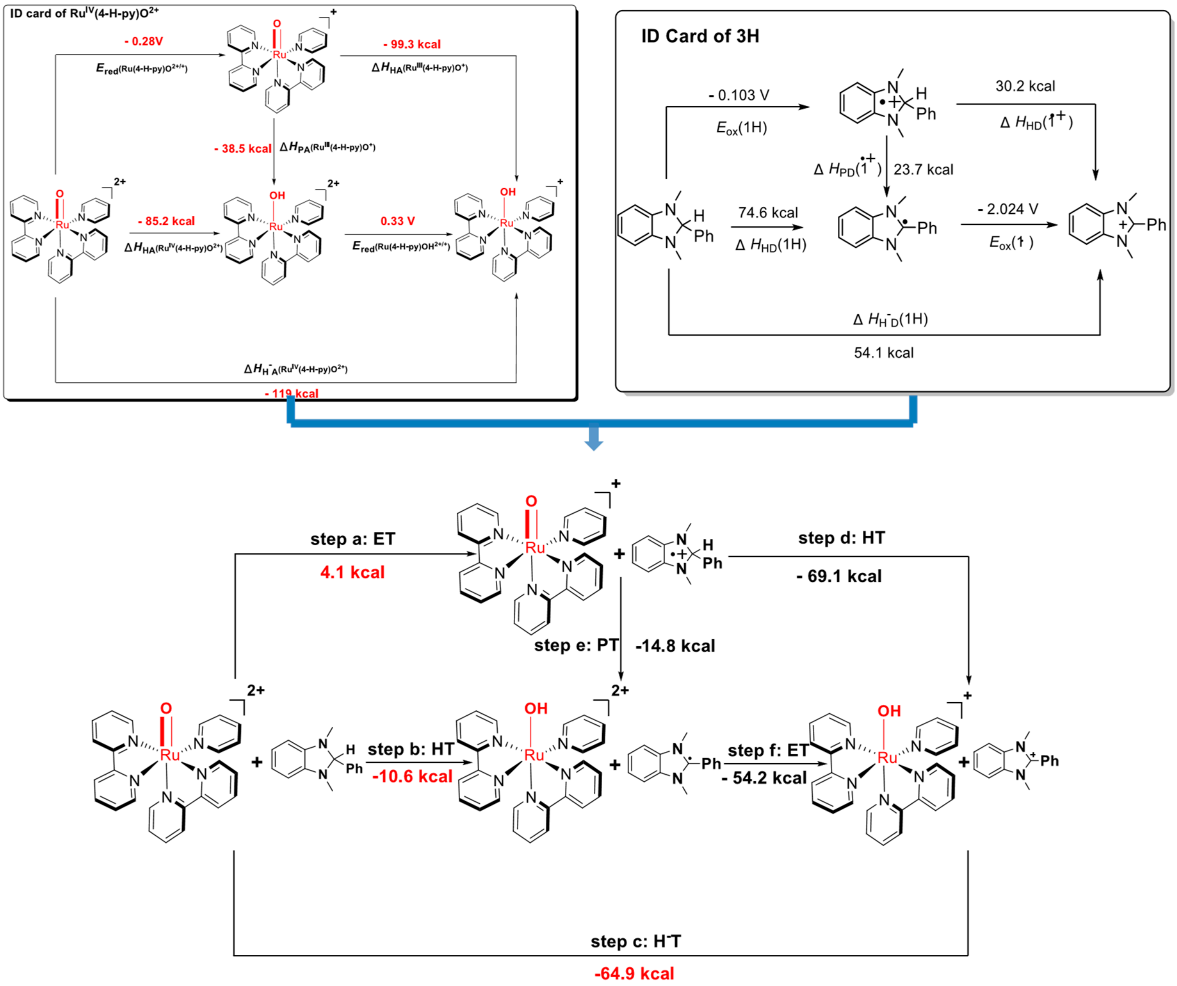

Figure S6. Thermodynamic analytic platform on the mechanism of hydride transfer from 3H to $[Ru^{IV}O]^{2+}$ at 298k in acetonitrile.

References

1. Sullivan, B.P.; Salmon, D.J.; Meyer, T.J. Mixed phosphine 2,2′-bipyridine complexes of ruthenium. *Inorg. Chem.* **1978**, *17*, 3334.

2. Sprintschnik, G.; Sprintschnik, H.W.; Kirsch, P.P.; Whitten, D.G. Photochemical reactions in organized monolayer assemblies. 6. Preparation and photochemical reactivity of surfactant ruthenium(II) complexes in monolayer assemblies and at water-solid interfaces. *J. Am. Chem. Soc.* **1977**, *99*, 4947–4953.
3. Moyer, B.A.; Meyer, T. J. Properties of the oxo/aqua system $(bpy)_2(py)RuO^{2+}/(bpy)_2(py)Ru(OH_2)^{2+}$. *Inorg. Chem.* **1981**, *20*, 436–444.
4. Dobson, J.C.; Helms, J.H.; Doppelt, P.; Sullivan, B.P.; Hatfield, W.E.; Meyer, T.J. Electronic structure of the oxidation catalyst cis(bipyridine)oxo(pyridine) ruthenium(IV) diperchlorate. *Inorg. Chem.* **1989**, *28*, 2200–2204.
5. Zhu, X.-Q.; Zhang, M.-T.; Yu, A.; Wang, C.-H.; Cheng, J.-P. Hydride, Hydrogen Atom, Proton and Electron Transfer Driving Forces of Various Five-membered Heterocyclic Organic Hydrides and Their Reaction Intermediates in Acetonitrile. *J. Am. Chem. Soc.* **2008**, *130*, 2501–2516.
6. Jacob, P.; Richter, W.; Ugi, I. 1,3,2λ5-Benzothiazaphosphole 2-Oxide and 1,3,2h5-Benzoxazaphosphole 2-Oxide Derivatives, New and Versatile Phosphorylating Reagents. *Liebigs Ann. Chem.* **1991**, *13*, 519–522.
7. Chikashita, H.; Komazawa, S.; Ishimoto, N.; Inoue, K.; Itoh, K. Nonacidic and Highly Chemoselective Protection of the Carbonyl Function. 3-Methylbenzothiazolines as a Base- and Acid-Resistant Protected Form for the Carbonyl Groups. *Bull. Chem. Soc. Jpn.* **1989**, *62*, 1215–1225.
8. Zhu, X.-Q.; Li, Q.; Hao, W.-F.; Cheng, J.-P. Dissociation Energies and Charge Distribution of the Co? NO Bond for Nitrosyl-α,β,γ,δ-tetraphenylporphinatocobalt(II) and Nitrosyl-α,β,γ,δtetraphenyl-orphinatocobalt(III) in Benzonitrile Solution. *J. Am. Chem. Soc.* **2002**, *124*, 9887−9893.
9. Zhu, X.-Q.; Hao, W.-F.; Tang, H.; Wang, C.-H.; Cheng, J.-P. Determination of N-NO Bond Dissociation Energies of N-Methyl-N-Nitrosobenzenesulfonamindes in Acetonitrile and Application in the Mechanism Analyses on NO Transfer. *J. Am. Chem. Soc.* **2005**, *127*, 2696–2708.
